# Enucleation for insulinoma: consolidating evidence through systematic review and meta-analysis

**DOI:** 10.1007/s00464-025-12099-0

**Published:** 2025-09-02

**Authors:** Marios Alogakos, Hayato Nakanishi, Dimitrios I. Athanasiadis, Soroush Farsi, Shreehari Suresh, Maria M. Witkowiak, Fatma A. M. Abdulsalam, Christian A. Than, Travis J. McKenzie, Eugene P. Ceppa

**Affiliations:** 1 School of Medicine, St George’s University of London, London, SW17 0RE UK; 2 School of Medicine, University of Nicosia, 2417 Nicosia, Cyprus; 3 Department of Surgery, Mayo Clinic, Rochester, MN 55905 USA; 4 Division of Surgical Oncology, Department of Surgery, Indiana University School of Medicine, 545 Barnhill Drive, EH 541, Indianapolis, IN 46202 USA; 5 Paul Foster School of Medicine, El Paso, TX 79905 USA; 6 Oxford University Hospitals NHS Foundation Trust, John Radcliffe Hospital, Headley Way, Headington, OX3 9DU UK; 7 Milton Keynes University Hospital, Milton Keynes, MK6 5LD UK; 8 School of Biomedical Sciences, The University of Queensland, St Lucia, 4072 Australia

**Keywords:** Insulinoma, Enucleation, Pancreas, Meta-analysis, Surgical outcomes

## Abstract

**Background:**

Pancreas-preserving procedures such as enucleation (EN) are indicated for select patients with insulinomas. Despite the increasing popularity of EN, no consensus has been reached on the preferred surgical approach for the management of insulinomas. The aim of this meta-analysis of proportions is to evaluate the safety and efficacy of EN for patients with pancreatic insulinoma.

**Methods:**

Cochrane, Embase, PubMed, Scopus, and Web of Science were searched from database inception to December 2023. The pooled mean and proportions were analyzed using a random-effects model. The review was registered prospectively with PROSPERO (CRD42024492786).

**Results:**

Twenty-one studies with 803 patients met the inclusion criteria. The pooled mean tumor diameter was 1.5 cm (95%CI: 1.3–1.6). The pooled mean operative time was 142 min (95%CI: 118–166), postoperative hospital stay was 9.5 days (95% CI: 7.2–11.7), and estimated blood loss (EBL) was 71.3 mL (95% CI: 47.3–95.3). The overall postoperative occurrence rate was 37.3% (95%CI: 0.264–0.481, *I*^2^ = 92%, *n* = 277), including 27% (95%CI: 0.179–0.360, *I*^2^ = 90%, *n* = 176) with any postoperative pancreatic fistula (POPF) and 1.5% (95%CI: 0.000–0.030, *I*^2^ = 0%, *n* = 4) with new-onset diabetes. Additionally, the pooled overall recurrence rate was 3.1% (95%CI: 0.016–0.045, *I*^2^ = 7%, *n* = 31), and the pooled rate of postoperative mortality was 1.1% (95%CI: 0.002–0.023, *I*^2^ = 0%, *n* = 6).

**Conclusion:**

EN appears safe and effective in managing pancreatic insulinoma for selected patients, with low rates of grade C POPF and recurrence. Despite the promising results, more selective criteria based on the location of insulinoma with a larger sample size and extended follow-up periods are necessary to ascertain the safety and efficacy of the treatment.

**Graphical Abstract:**

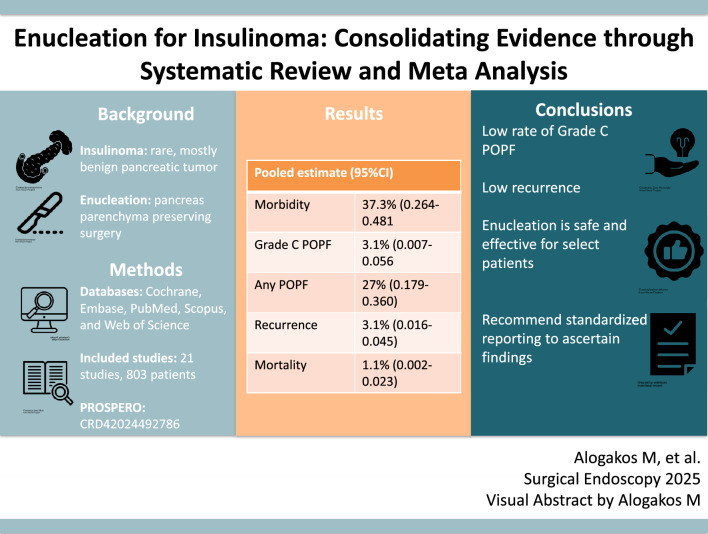

**Supplementary Information:**

The online version contains supplementary material available at 10.1007/s00464-025-12099-0.

## Introduction

Insulinoma is a rare type of pancreatic neuroendocrine tumor that affects 1–2 per million patients worldwide, with an incidence peak in the fifth decade of life [1]. Approximately, ninety percent of insulinomas are characterized as a benign, single-focal islet cell adenoma and represent the most frequently occurring functional neuroendocrine tumor of the gastrointestinal tract [1]. When evaluating the surgical approach for insulinoma requiring operation, preoperative localization of the tumor with high-resolution imaging and endoscopic ultrasound is crucial [2]. Currently, there is no consensus on the selection criteria or the optimal surgical procedure for managing symptomatic insulinomas.

Traditionally, surgical approaches for patients with insulinomas consist of standardized pancreatectomy (SP), including pancreatoduodenectomy (PD) and distal pancreatectomy (DP). Although SP can now be completed more safely, it is still associated with high morbidity and long-term morbidity such as delayed gastric emptying (DGE), exocrine, and endocrine pancreatic insufficiency [3].

To overcome the limitation of SP, pancreas-preserving resections such as enucleation (EN) have been proposed as an alternative surgical option for selected patients. Insulinoma EN is favored for its ability to preserve healthy pancreatic tissue and minimize the risk of postoperative pancreatic insufficiency [4, 5]. However, EN itself carries a high risk of postoperative morbidity and is not commonly recommended when the tumor is deeply embedded in the pancreatic parenchyma or near the main pancreatic duct (MPD) [6]. To mitigate the risk, Okabayashi et al. theorized that a minimally invasive approach with intraoperative ultrasound (IOUS), despite not being available at all centers, could yield more optimal perioperative outcomes [7].

The latest North American Neuroendocrine Tumor Society (NANETS) guidelines suggested that EN should be used for smaller tumors, particularly those that are likely benign and located more than 2–3 mm from the MPD [8]. Nonetheless, most studies on EN for insulinomas have been reported at single institutions, and as a result, robust evidence regarding EN for insulinomas is lacking. To our knowledge, no existing meta-analyses have been performed as an analysis of proportions on EN for insulinoma, specifically addressing postoperative outcomes and morbidity. Therefore, this meta-analysis aims to assess the safety and efficacy of EN for insulinomas by synthesizing the available literature, thereby guiding best clinical practices.

## Methods

### Search strategy and data sources

A comprehensive search of several databases from each database’s inception to December 19th, 2023 was conducted. The databases searched included Cochrane, Embase, PubMed, Scopus, and Web of Science. This systematic review was reported in accordance with the PRISMA 2020 reporting standards [9]. The search strategy was designed and conducted by an experienced librarian with input provided by the research team. Controlled vocabulary supplemented with keywords was searched for adult patients who underwent EN for pancreatic insulinoma. The actual strategy listing all search terms used and how they were combined is available in Supplementary Item I. The review was registered prospectively with PROSPERO (CRD42024492786).

### Eligibility criteria and risk of bias assessment

Eligible studies were randomized controlled trials (RCTs) or cohort studies that met the following inclusion criteria: 1) adult participants who underwent EN for insulinoma; 2) outcomes of postoperative overall complication rates; 3) outcomes of postoperative recurrence rates; and/or 4) sample size of at least 10 insulinoma EN cases. Case reports, case series, abstracts, reviews, conference abstracts, and articles that were not reported in English were excluded from the study. Lastly, this meta-analysis also excluded studies if EN was not the only surgical intervention employed, and the eligibility of studies was assessed based on per-protocol analysis. The critical appraisal for each study, including assessing for risk of bias, was independently evaluated by two authors (SS and MA) using the Joanna Briggs Institute (JBI) Critical Appraisal Checklist for Case Series [10]. Studies were considered to have low risk of bias if at least 8 items were rated “Yes,” moderate risk if 5 to 7 items were rated “Yes,” and high risk if fewer than 5 items were rated “Yes.” Any discrepancies were discussed by the two independent assessors, with disagreements addressed via an adjudicator (HN). Results of the risk of bias assessment of all included studies are shown in Supplementary Item II. Four independent assessors (MA, SS, SF, DIA) conducted article screening and data extraction. Any disagreements were discussed with co-authors and adjudicated by CAT. The certainty of evidence was evaluated using the Grading of Recommendations, Assessment, Development, and Evaluation (GRADE) approach, with priori thresholds set for small, moderate, and large effects [11]. The results are summarized in Supplementary Item III.

### Extracted outcomes

For baseline characteristics, the following were extracted: location and diameter of the tumor, the American Society of Anesthesiologists (ASA) score, and the World Health Organization (WHO) classification of endocrine tumors. For perioperative outcomes, the following were extracted: operative approach, operative time, estimated blood loss (EBL), length of hospital stays, and complication rates. For postoperative outcomes, the following were extracted: readmission, reoperation rates, recurrence, immediate new-onset diabetes, exocrine pancreatic insufficiency, and mortality rates.

### Insulinoma (Enucleation) diagnostic criteria

The inclusion criteria for insulinoma diagnosis varied across studies, with nine studies [6, 19, 20, 22, 24, 26, 28, 32, 36] relying on biochemical evidence of hypoglycemia and hyperinsulinemia, while six studies [6, 20, 21, 21–32, 36] relied on a 72-h fasting test. Across three studies [23, 28, 32], the presence of Whipple’s triad or a plasma insulin-to-glucose ratio greater than 0.3 were used as inclusion criteria. Additionally, two studies [34, 35] required confirmation through imaging techniques, such as computed tomography (CT) or magnetic resonance imaging (MRI), while three studies [27, 28, 33] relied on histopathological verification post-surgery. Twelve studies [6, 22, 24–29, 31, 33, 35, 36] reported utilizing IOUS to evaluate tumor morphology and its proximity to the main pancreatic duct while preoperative CT, MRI, and intraductal ultrasound were also utilized but with much less frequency. None of the included studies reported on the use of preoperative biopsy. The inclusion criteria for EN across eight studies [6, 19, 21–23, 27, 31, 33] centered on the size and location of the tumor, as well as proximity to critical pancreatic structures. The EN approach was primarily selected for small, superficial, benign tumors, particularly when they were at least 2 mm from the MPD and adjacent vasculature.

### POPF grades and DGE criteria

Postoperative pancreatic fistula (POPF) was classified into three grades of increasing severity according to the International Study Group for Pancreatic Surgery (ISGPS) grading system. A biochemical leak, previously categorized as Grade A, is characterized by asymptomatic drainage of pancreatic fluid rich in amylase, from drains. Grade B requires a change in the postoperative management and specific treatments, such as parental or enteral nutrition and antibiotics, to promote the healing of the fistula. Grade C involves major deviation from the expected clinical course and the need for invasive procedures including surgical reoperation [12]. Across two studies, DGE was defined based on the ISGPS classification or as either the maintenance of a nasogastric tube (NGT) or recurrent postprandial vomiting on postoperative day (POD) 10 [31, 33]. The latest ISGPS classification from 2017, defined DGE as the use of NGT for at least 3 days postoperatively, the need to reinsert the NGT for persistent vomiting after POD 3, or failure to resume oral diet by POD 7 [13]. Although the ISGPS classification has been updated overtime, the core criteria remain focused on prolonged NGT use, NGT reinsertion postoperatively, or extended intolerance of solid foods.

### Statistical analysis

The pooled means and proportions of our data were analyzed using a random-effects, generic inverse variance method of DerSimonian and Laird, which assigns the weight of each study based on its variance [14]. The heterogeneity of effect size estimates across the studies was quantified using the Q statistic and *I*^2^ (P < 0.10 was considered significant). A value of *I*^2^ of 0–25% indicates insignificant statistical heterogeneity, 26–50% low heterogeneity, and 51–100% high heterogeneity [15]. Furthermore, a leave-one-out sensitivity analysis was conducted to assess each study’s influence on the pooled estimate by omitting one study at a time and recalculating the combined estimates for the remaining studies. Publication bias was assessed visually using a funnel plot, as depicted in Supplementary Item IV [16]. If mean and standard deviation (SD) were unavailable, the median was converted to mean using the formulas from the Cochrane Handbook for Systematic Reviews of Interventions [17]. Data analysis was performed using Open Meta analyst software (CEBM, Brown University, Providence, Rhode Island, USA).

## Results

### Study selection and patient characteristics

The initial literature search yielded 991 potentially relevant articles, of which twenty-one unique studies involving 803 patients were included in this meta-analysis [6, 18–37]. All the selected studies were retrospective, with six [18, 19, 22, 29, 32, 33] being multicenter and the remaining fifteen [6, 20, 21, 23–28, 30, 31, 34–37] conducted at a single-center series. The reported mean age ranged from 22 to 50 years, and 62.2% of patients were females. A PRISMA flowchart of the study selection process is depicted in Supplementary Item V. The baseline characteristics of the included studies are described in Table 1.

**Table 1:** Baseline characteristics of included studies

Study	Publication Year	Country	Study Type	Number of Centers (N)	Total Participants (N)	Gender (Female), N (%)	Age, Mean ± SD (Years)	BMI mean ± SD (kg/m2)	Length of Follow-up, Mean ± SD (Months)
Ayav et al. [18]	2005	France	Retrospective	Multicenter	19	NR	NR	NR	NR
Belfiori et al. [19]	2018	Italy	Retrospective	Multicenter	71	45	47	24.65 ± 8.5	74 ± 40.3
Chen et al. [20]	2002	China	Retrospective	Single center	59	NR	NR	NR	NR
Chirletti et al. [21]	2000	Italy	Retrospective	Single center	26	NR	NR	NR	NR
Crippa et al. [22]	2012	Italy	Retrospective	Multicenter	106	NR	NR	NR	NR
Geoghegan et al. [23]	1994	UK	Retrospective	Single center	18	NR	NR	NR	NR
Guo et al. [24]	2014	China	Retrospective	Single center	32	NR	NR	NR	NR
Liu et al. [25]	2007	China	Retrospective	Single center	32	NR	NR	NR	NR
Luo et al. [26]	2009	China	Retrospective	Single center	18	NR	NR	NR	NR
Menegaux et al. [27]	1993	France	Retrospective	Single center	14	NR	NR	NR	NR
Naples et al. [28]	2022	USA	Retrospective	Single center	25	NR	NR	NR	NR
Nikfarjam et al. [6]	2008	USA	Retrospective	Single center	21	NR	NR	NR	NR
Peltola et al. [29]	2018	Finland	Retrospective	Multicenter	31	NR	NR	NR	NR
Spelsberg et al. [30]	1979	Germany	Retrospective	Single center	13	NR	NR	NR	NR
Tsang et al. [31]	2016	Hong Kong	Retrospective	Single center	18	NR	NR	NR	NR
van Beek et al. [32]	2020	Netherlands	Retrospective	Multicenter	20	NR	NR	NR	83.4 ± 81.8
Vezzosi et al. [33]	2015	France	Retrospective	Multicenter	18	12	22	NR	78 ± 99.5
Wei et al. [34]	2016	China	Retrospective	Single center	19	15	45.5	27.5 ± 5.7	NR
Xu et al. [35]	2021	China	Retrospective	Single center	81	44	50	NR	NR
Yin et al. [36]	2023	China	Retrospective	Single center	33	22	46.3	26.9 ± 3.2	NR
Zhang et al. [37]	2012	China	Retrospective	Single center	129	NR	NR	NR	NR

*BMI* Body mass index, *NR* Not reported, *SD* Standard deviation

### Risk of bias assessment and certainty of evidence

The results of the risk of bias assessment of all studies included are shown in Supplementary Item II. Eight studies [6, 19, 22, 24, 28, 29, 31, 34] were judged to have low risk of bias. Eleven studies [20, 21, 23, 25–27, 32, 33, 35–37] were judged to have moderate risk of bias with potential confounding and selection bias due to inconsistent reporting of baseline characteristics, multicenter design, and prolonged study periods. Two studies [18, 30] were judged to have high risk of bias due to unclear inclusion criteria and lack of appropriate adjustment for confounding factors. Nonetheless, all the studies included were deemed adequate within the selection domain. The results demonstrating the certainty of evidence are summarized in a GRADE evidence table in Supplementary Table III.

### Clinical characteristics

**Fig. 1:**
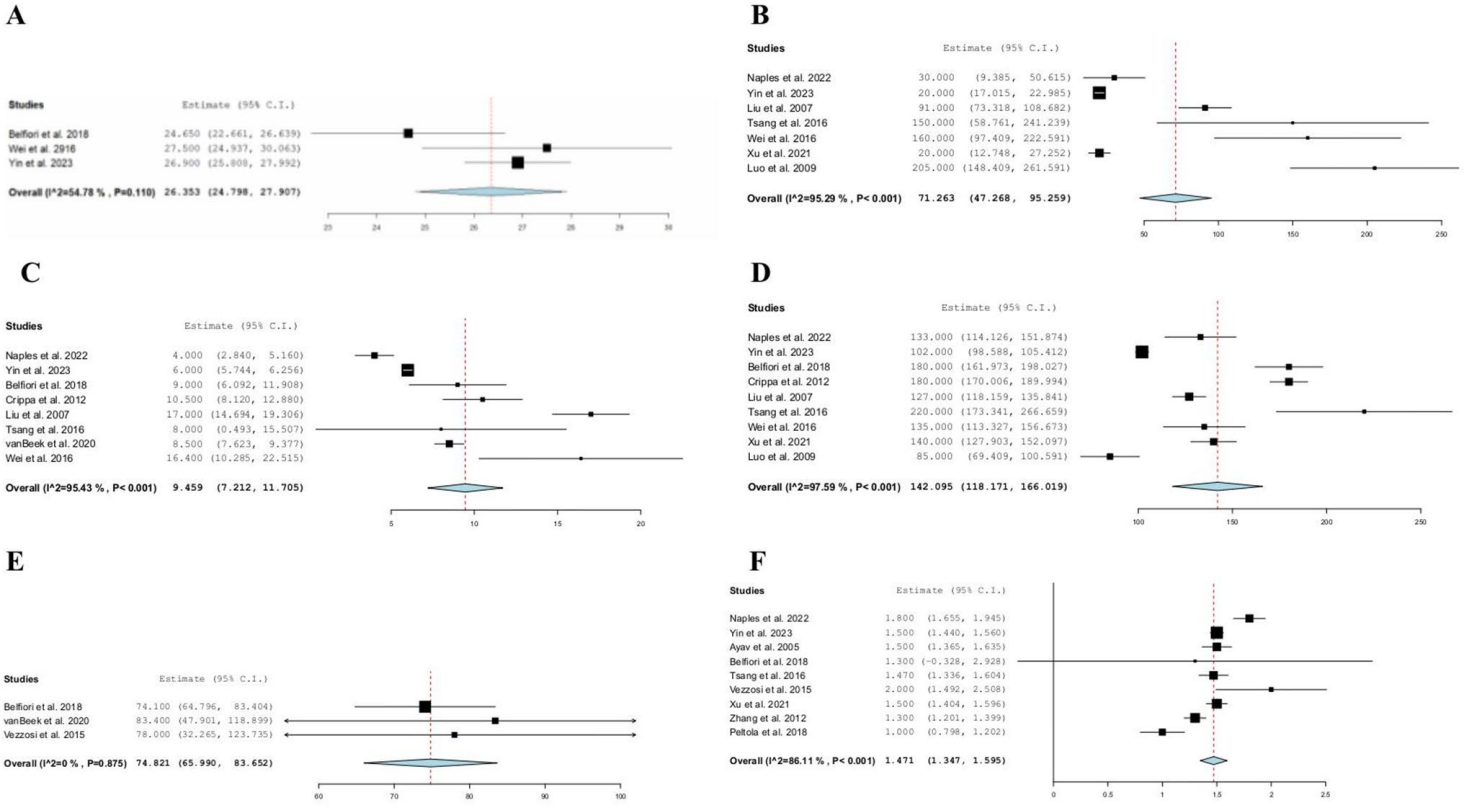
The pooled patient characteristics

Three studies [19, 34, 36] reported the preoperative body mass index (BMI) with a pooled mean of 26.3 kg/m^2^ (95% CI: 24.7–27.9, *I*^2^ = 54%). Fourteen studies [6, 18, 19, 22, 24–26, 28, 31, 33–37] reported the operative approach, consisting of open (69.8%, *n* = 369), laparoscopic (23.3%, *n* = 123), and robotic surgery (6.8%, *n* = 36). The mean preoperative ASA score was 2.1 in two studies [34, 36]. The pooled mean follow-up period was 74 months (95% CI: 65.9–83.6, *I*^2^ = 0%), with the longest follow-up period being 83 months. Clinical characteristics of our included studies are summarized in Table 2 and Fig. 1.

**Table 2:** Clinical characteristics of included studies

Study	Surgical approach			Tumor Diameter Mean ± SD (cm)	Operative time Mean ± SD (min)	Hospital stay Mean ± SD (days)	Estimated blood loss Mean ± SD (mL)
	Open	Laparoscopic	Robotic				
Ayav et al. [18]	NR	19	NR	1.5 ± 0.37	NR	NR	NR
Belfiori et al. [19]	56	12	3	1.3 ± 7	180 ± 77.5	9 ± 12.5	NR
Chen et al. [20]	NR	NR	NR	NR	NR	NR	NR
Chirletti et al. [21]	NR	NR	NR	NR	NR	NR	NR
Crippa et al. [22]	100	6	0	1.45	180 ± 52.5	10.5 ± 12.5	NR
Geoghegan et al. [23]	NR	NR	NR	NR	NR	NR	NR
Guo et al. [24]	NR	4	0	NR	NR	18	NR
Liu et al. [25]	26	6	0	1.4 ± 0.29	127 ± 23	17 ± 6	91 ± 46
Luo et al. [26]	4	16	0	NR	85	NR	205 ± 122.5
Menegaux et al. [27]	NR	NR	NR	NR	NR	NR	NR
Naples et al. [28]	15	10	NR	1.8 ± 0.37	133 ± 48.1	4 ± 2.96	30 ± 52.59
Nikfarjam et al. [6]	20	1	0	NR	NR	NR	NR
Peltola et al. [29]	NR	NR	NR	1 ± 0.57	NR	NR	NR
Spelsberg et al. [30]	NR	NR	NR	NR	NR	NR	NR
Tsang et al. [31]	12	6	0	NR	220 ± 201	8 ± 16.2	150 ± 197.5
van Beek et al. [32]	NR	NR	NR	NR	133	8.5 ± 2	50
Vezzosi et al. [33]	13	5	0	2 ± 1.1	NR	NR	NR
Wei et al. [34]	19	0	0	NR	135 ± 48.2	16.4 ± 13.6	160 ± 139.2
Xu et al. [35]	13	NR	NR	1.5 ± 0.44	140 ± 55.5	NR	20 ± 33.3
Yin et al. [36]	NR	NR	33	1.5 ± 0.17	102 ± 10	6 ± 0.75	20 ± 8.75
Zhang et al. [37]	91	38	NR	1.3 ± 0.57	NR	NR	NR

*NR* Not reported, *POPF* Postoperative pancreatic fistula, *SD* Standard deviation

### Tumor characteristics

Eleven [18, 19, 21, 24–27, 30, 33–35] studies reported anatomic location of the pancreatic insulinoma: head (45.0%, *n* = 136), body (30.8%, *n* = 93), tail (18.5%, *n* = 56), and neck (5.6%, *n* = 17). Ten studies [18, 19, 22, 25, 28, 29, 33, 35–37] reported tumor diameter, with a pooled mean diameter of 1.5 cm (95%CI: 1.3–1.6, *I*^2^ = 86%). The histological classification of insulinoma among three studies [31, 35, 36] consisted of G1 (63.0%, *n* = 80), G2 (36.2%, *n* = 46), and G3 (0.8%, *n* = 1). The tumor characteristics of included studies are comprehensively described in in Table 2.

### Postoperative outcomes

**Fig. 2:**
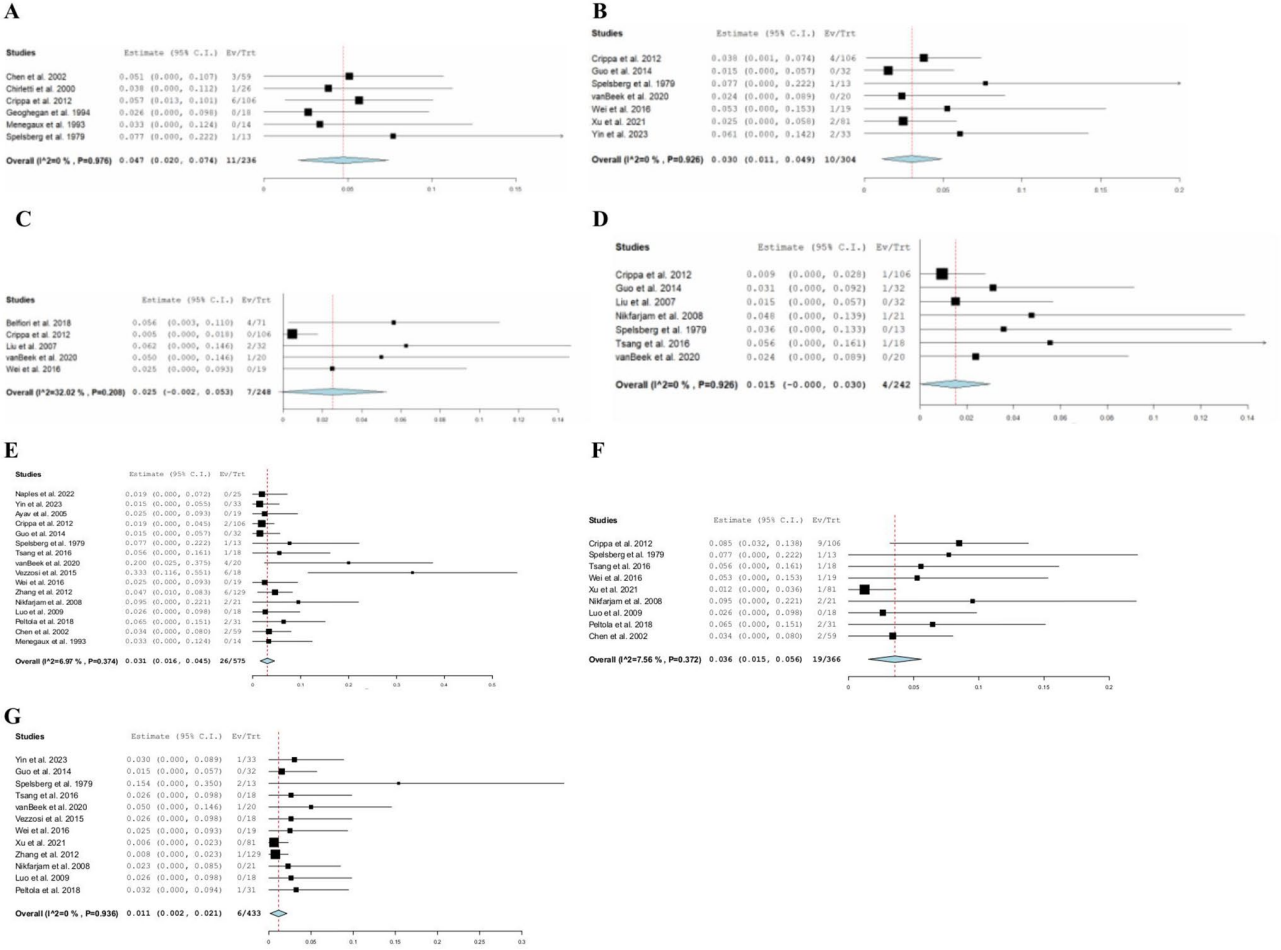
The pooled postoperative outcomes

Of 20 studies [6, 18–35, 37], overall postoperative morbidity after insulinoma EN was 37.3% (95%CI: 0.264–0.481, *I*^2^ = 92%, *n* = 277). The most commonly reported morbidities were POPF (27%, *n* = 176), abdominal collection (3.3%, *n* = 21), acute pancreatitis (1.7%, *n* = 11), and postoperative hemorrhage (1.7%, *n* = 10). Among 7 studies [6, 22, 24, 25, 30–32], 4 patients (1.5%) had immediate new-onset diabetes (95%CI: 0.000–0.030, *I*^2^ = 0%). Among the 6 studies [20–23, 27, 30] reporting on postoperative acute pancreatitis (POAP), 11 patients (5%) were diagnosed with POAP (95% CI: 0.020–0.074, *I*^2^ = 0%). Across 7 studies [22, 24, 30, 32, 34–36], 10 patients (3%) experienced postoperative hemorrhage (95% CI: 0.011–0.049, *I*^2^ = 0%). DGE was reported in 5 studies [19, 22, 25, 32, 34] with a total of 7 patients (3%) affected (95% CI: 0.002–0.053, *I*^2^ = 0%). Across 5 studies [19, 26, 28, 31, 36], the rate of conversion to open surgery due to unfavorable tumor location was 7%, *n* = 12. The pooled postoperative outcomes are shown in Fig. 2.

**Fig. 3:**
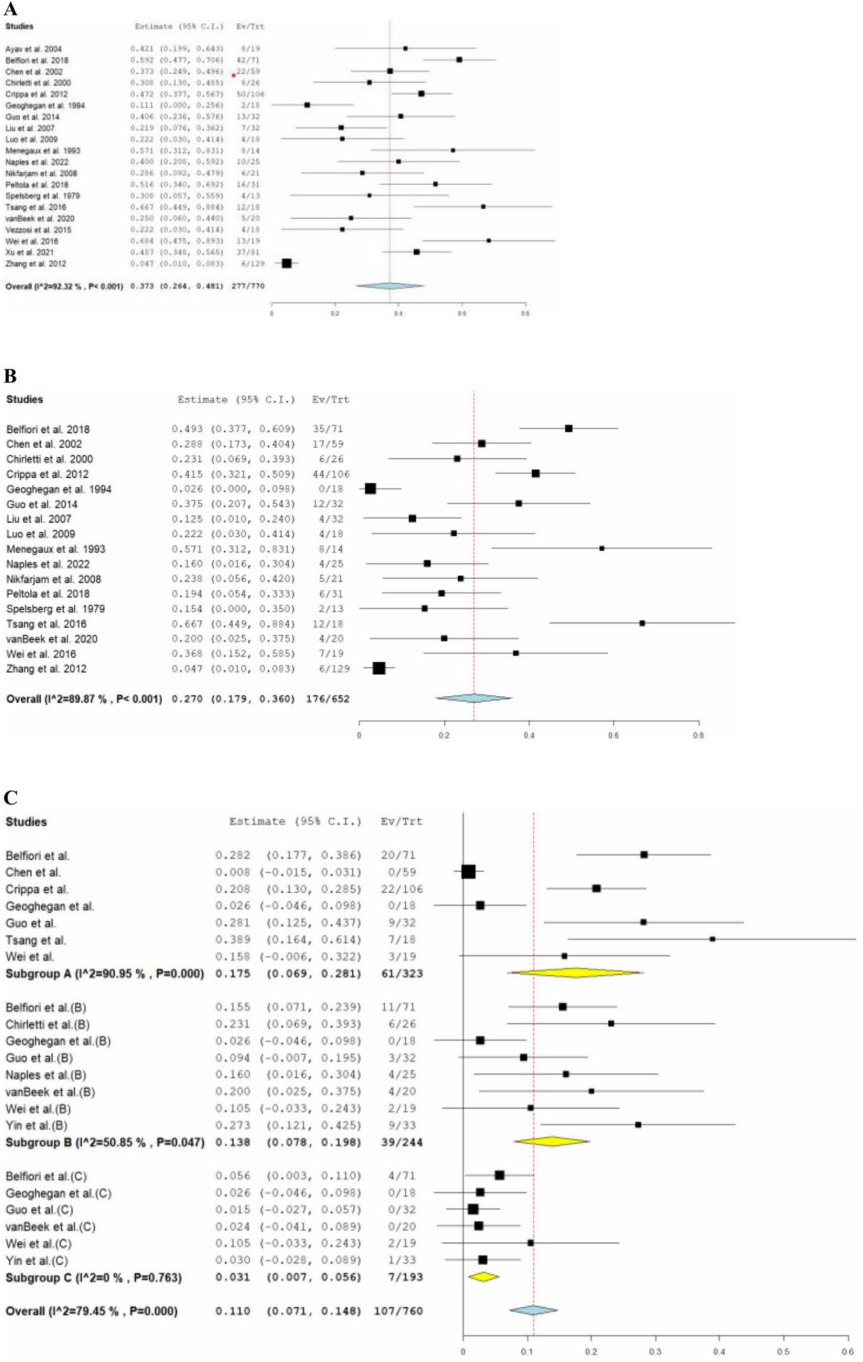
The pooled estimate postoperative outcomes

The pooled overall rate of POPF across seventeen studies [6, 19–32, 34, 37] was 27% (95% CI: 0.179–0.360, *I*^2^ = 90%, *n* = 176). Subgroup analysis of POPF, across 11 studies [19–24, 28, 31, 32, 34, 36], demonstrated 17.5% (95% CI: 0.069–0.281, *I*^2^ = 95%, *n* = 61) with a biochemical leak, 13.8% (95% CI: 0.078–0.198, *I*^2^ = 51%, *n* = 39) with Grade B, and 3.1% (95% CI: 0.007–0.056, *I*^2^ = 0%, *n* = 7) with Grade C. The pooled mean operative time was 142 min (95%CI: 118–166, *I*^2^ = 98%), and the pooled mean hospital stay was 9.5 days (95% CI: 7.2–11.7, *I*^2^ = 95%). Eight studies [25, 26, 28, 31, 32, 34–36] reported EBL with a reported pooled mean of 71.3 mL (95% CI: 47.3–95.3, *I*^2^ = 95%). The postoperative mortality was 1.1% (95%CI: 0.002–0.023, *I*^2^ = 0%, *n* = 6) among the 15 studies [6, 18, 19, 21, 24, 26, 29–37], and recurrence rate was 3.1% (95%CI: 0.016–0.045, *I*^2^ = 7%, *n* = 31) among 17 studies [6, 18–20, 22, 24, 26–34, 36, 37]. Among 8 studies [6, 19, 20, 22, 29, 31–33], recurrence consisted of local recurrence (0.7%, *n* = 5), tumor persistence (0.3%, *n* = 2), distant metastasis (0.6%, *n* = 4), multifocal tumors (0.3%, *n* = 2), and MEN-1 related (1.7%, *n* = 11). Reoperation rate among 10 studies [6, 19, 20, 22, 26, 29–31, 34, 35] was 3.6% (95% CI: 0.025–0.056, *I*^2^ = 25%, *n* = 26). The postoperative outcomes of included studies are comprehensively described in Table 3. The pooled estimate postoperative adverse events are shown in Fig. 3 and a summary of overall postoperative complications following insulinoma EN is described in Supplementary Item VI.

**Table 3:** Postoperative outcomes of included studies

Study	Biochemical leak N, (%)	POPF B N, (%)	POPF C N, (%)	Overall POPF N, (%)	Postoperative complication N, (%)	Recurrence N, (%)	Reoperation N, (%)	Readmission N, (%)	Overall mortality N, (N)
Ayav et al. [18]	NR	NR	NR	NR	8 (42%)	0	NR	0	0
Belfiori et al. [19]	20 (28.2%)	11 (15.5%)	4 (5.6%)	35 (49.3%)	42 (59%)	4 (5.6%)	7 (9.9%)	9 (12.7%)	0
Chen et al. [20]	0	NR	NR	17 (28.8%)	22 (37%)	2 (3.3%)	2 (3.9%)	NR	NR
Chirletti et al. [21]	NR	6 (23.1%)	NR	6 (23.1%)	8 (31%)	NR	NR	1 (3.8%)	1 (3.8%)
Crippa et al. [22]	22 (20.8%)	NR	NR	44 (41.5%)	50 (47%)	2 (1.8%)	9 (8.5%)	4 (3.8%)	NR
Geoghegan et al. [23]	0	0	0	0	2 (11%)	NR	NR	NR	NR
Guo et al. [24]	9 (28.1%)	3 (9.4%)	0	12 (37.5%)	13 (41%)	0	NR	NR	0
Liu et al. [25]	NR	NR	NR	4 (12.5%)	7 (27%)	NR	NR	NR	NR
Luo et al. [26]	NR	NR	NR	4 (22.2%)	4 (25%)	0	0	NR	0
Menegaux et al. [27]	NR	NR	NR	8 (57.1%)	8 (57%)	0	NR	NR	NR
Naples et al. [28]	NR	4 (16%)	NR	4 (16%)	10 (40%)	0	NR	NR	NR
Nikfarjam et al. [6]	NR	NR	NR	5 (23.8%)	6 (29%)	2 (9.5%)	2 (9.5%)	NR	0
Peltola et al. [29]	NR	NR	NR	6 (19.4%)	16 (52%)	2 (9.4%)	2 (6.5%)	NR	1 (3.2%)
Spelsberg et al. [30]	NR	NR	NR	2 (15.4%)	4 (31%)	1 (7.6%)	1 (7.7%)	NR	2 (15.4%)
Tsang et al. [31]	7 (38.9%)	NR	NR	12 (66.7%)	12 (67%)	2 (11.1%)	1 (5.6%)	NR	0
van Beek et al. [32]	NR	4 (20%)	0	4 (20%)	5 (25%)	4 (20%)	NR	4 (20%)	1 (5%)
Vezzosi et al. [33]	NR	NR	NR	NR	4 (22%)	6 (33.3%)	NR	NR	0
Wei et al. [34]	3 (15.79%)	2 (10.5%)	2 (10.5%)	7 (36.8%)	13 (68%)	0	1 (5.3%)	NR	0
Xu et al. [35]	NR	NR	NR	NR	37 (46%)	NR	1 (1. 2%)	NR	0
Yin et al. [36]	23 (69.7%)	9 (27.3%)	1 (3%)	33 (100%)	100%	0	NR	NR	1 (3%)
Zhang et al. [37]	NR	NR	NR	6 (4.65%)	6 (5%)	6 (1.6%)	NR	NR	1 (0.8%)

*NR* Not reported, *SD* Standard deviation

## Discussion

Successful surgical management of insulinoma requires significant expertise to minimize the risk of postoperative morbidity. Debates on the selection criteria for pancreatic EN are ongoing, and evaluation of the perioperative outcomes is critical for surgeons to undertake the surgical management of insulinoma. The primary aim of this meta-analysis was to investigate the safety and efficacy of EN for patients with pancreatic insulinoma. Based on the 21 included studies, this meta-analysis revealed a notable morbidity rate with high overall incidence of POPF after EN. Conversely, our study demonstrated low incidence of grade C POPF, mortality, recurrence, and reoperation rates.

Establishing proper indications for EN remains critical to minimize morbidity, yet criteria vary across studies and are often debated. At the present, EN is generally recommended for small (< 2 cm) benign functional tumors located away from the MPD [38]. In contrast, EN is typically avoided for larger tumors due to concerns regarding malignant potential, inadequate resection margins, limited lymph node clearance, and an increased risk of clinically relevant POPF [3]. A tumor distance greater than 2–3 mm is preferable to minimize the risk of POPF [39]. Additional risk factors include a soft pancreas or a small pancreatic duct (≤ 3 mm), which may increase the risk of POPF or leak due to tissue fragility [40]. Moreover, pancreatic ducts located near the posterior edge of the pancreatic stump are also at higher risk, even in cases where the pancreas is thick or wide [41]. Current clinical practice for managing neuroendocrine tumors often depends on the surgeon’s discretion due to the lack of clear guidelines.

Anatomic considerations are important when selecting the optimal surgical approach for insulinoma. EN is often preferred for small insulinomas in the pancreatic head because it is less invasive and preserves normal pancreatic tissue along with physiologic function [40]. This aligns with our finding that 45% of insulinomas were located at the pancreatic head. However, because the pancreatic head is close to the bile duct and the duodenum, there is a higher risk of morbidity, such as POPF and bile duct injury [42]. Jilesen *et al*. identified pancreatic head location as an independent risk factor for POPF. In their study, 31% of patients undergoing EN developed a POPF and in 40% of these cases, the tumor resided in the pancreatic head [42]. Despite the increased risk, EN is commonly used for lesions in this area due to its lower-risk profile compared to PD, which is associated with additional serious morbidities like DGE and anastomotic leaks [42]. In contrast, insulinomas in the pancreatic tail are typically treated with DP, which is generally associated with lower serious morbidity because of the pancreatic tail’s distance from critical structures [40]. While EN is preferred for small, benign insulinomas in both the pancreatic head and tail, the pancreatic head often requires more careful consideration due to its proximity to critical anatomy. Future studies should stratify POPF rates by anatomical location and their proximity to the MPD, as surgical indications vary accordingly.

This meta-analysis is the first to evaluate POPF after EN with stratification by POPF grade. Our study observed high biochemical leak and Grade B POPF, with low incidence of grade C POPF, consistent with the previous meta-analysis by Hüttner *et al*. [43]. Hackert *et al*. attributed the high incidence of POPF to non-anatomic resections that expose the pancreatic wound near the MPD [39, 44]. However, they claimed that these fistulas are often low-grade and do not greatly impact long-term outcomes. Conversely, Li *et al*. observed no incidence of grade C POPF cases after EN and argued that the MPD should no longer be the restricted site for EN, when targeted intraoperative strategies are utilized in high-risk cases. These include MPD-centered dissection with pre-placed support tubes, IOUS for assessing tumor-duct proximity, and reinforcement of the pancreatic wound with a ligamentum teres hepatis flap [45].

One proposed method to reduce POPF-related morbidity is the preoperative placement of a pancreatic stent at the time of endoscopic retrograde pancreatography (ERCP) [46]. In a study by Xu *et al*., 37.5% of stented high-risk patients developed grade B or C POPF, compared to 71.4% of non-stented patients [35]. However, stenting carried additional risks such as post-ERCP pancreatitis [39]. Giuliani *et al*., reported postoperative complications in 7 out of 10 patients who underwent pancreatic stenting [47]. Currently, a prospective multicenter study is investigating the role of preoperative stenting in insulinoma EN of the head and neck [48]. An alternative approach involves using a round hepatic ligament flap to provide peritoneal coverage over the EN site. In its initial description by Hackert *et al*. in 2013, none of the 7 patients developed POPF. However, its use is limited in cases involving the pancreatic tail, where the ligament might not reach despite mobilization [49]. Another proposed strategy is the prophylactic use of somatostatin analogs to reduce POPF by decreasing pancreatic secretions and pancreatic ductal pressure [45, 49]. Although several studies support this approach, meta-analyses have revealed conflicting evidence regarding their effectiveness [50–52]. The latest NANETS guidelines do not routinely recommend octreotide. As an alternative, Pasireotide may reduce clinically significant POPF, although its use is limited by side effects and high cost [53, 54]. To date, despite various attempts to address the issue, POPF remains one of the most significant challenges in EN, particularly in neuroendocrine tumor patients [41].

A growing body of evidence suggests that minimally invasive surgery (MIS) offers several distinct advantages over the open approach, although some limitations remain. Clinically, studies by Belfiori *et al*. and Lopez *et al*. consistently showed reduced intraoperative blood loss, shorter operative times, and quicker recovery [19, 55]. This is corroborated by a meta-analysis by Roesel *et al*., which reported comparable or improved short-term outcomes with MIS, including shorter hospital stays and fewer major complications [56]. Robotic platforms may enhance these benefits by improving dexterity, precision, and visualization, although increased cost and operative times remain barriers to widespread adoption [57]. Importantly, IOUS plays a critical role in MIS where tactile feedback is absent. As emphasized by Cunha *et al*. and Fernandez *et al*., laparoscopic ultrasonography facilitates precise tumor localization and assessment of proximity to the MPD, vital for minimizing POPF risk [58, 59]. However, tumors near the MPD or in deep parenchymal locations may still necessitate conversion to open surgery, especially for lesions in the head, where open surgery remains the standard [59]**.** This is supported by the surgeon’s ability during open surgery to palpate the pancreas and assess the tumor’s relationship to the duct in real time, along with the greater feasibility of performing ductal repair, stenting, or complex reconstruction if the MPD is injured [56]. Nonetheless, MIS remains a safe and technically refined option in select cases, particularly when guided by preoperative imaging and IOUS.

This meta-analysis confirms that despite a high rate of POPF, pancreatic EN may offer benefits over SP, including faster postoperative recovery, shorter surgery times, and reduced EBL, due to the less invasive nature of the procedure [22, 32, 40, 60]. It can be hypothesized that these findings are due to EN's minimal disruption and preservation of pancreatic function, compared to DP’s more extensive tissue and vessel dissection [22, 32, 40, 60]. Moreover, observed recurrence rates post-EN were low, with 4.1% to 5.6% for benign insulinomas [19, 61]. However, the few cases of recurrence may be due to inadequate lymph node sampling during EN, leading to tumor under staging and reduced treatment efficacy [22]. While RCTs investigating EN against SP could build upon these findings, it is understandably challenging given the rarity of insulinomas.

When assessing the POPF rate for EN of pancreatic insulinoma, comparison with SP is essential. Pedrazzili *et al.* reported a 3.5% incidence of grade C POPF in a systematic review of PD [62]. Interestingly, both Tsang *et al*. and Crippa *et al*. have shown no differences between patients who underwent EN and pancreatectomy [22, 31]. On the other hand, Heidsma *et al*. found twofold incidence of grade B/C POPF in the EN group compared with PD or DP [40]. Radiofrequency ablation (RFA) has emerged as a promising alternative to surgery for insulinomas, especially in high-risk surgical candidates [63]. While RFA has been shown to be effective in controlling hypoglycemia and reducing tumor burden, it is associated with a higher local recurrence rate of 16.9% [64, 65]. Despite its hypothesized advantages, such as ease of use, reduced severe morbidity, and lower cost, RFA poses challenges, particularly with tumors near mesenteric vessels, the MPD or superficial tumor sites, leading to complications such as stenosis, fistula formation, or duodenal injury [63, 64, 66]. At the present, the existing data do not support this as a primary therapy for the management of insulinoma.

As with all meta-analyses, limitations are present. The main limitation of this meta-analysis is the design of the studies, as the majority were retrospective and observational designs rather than randomized controlled trials. This led to inherent biases, such as selection and information bias as well as confounders, which must be considered when assessing the outcomes of this meta-analysis, particularly given the lack of stratification in each instance of POPF [67]. To illustrate, some studies might have underreported the occurrence of POPF, postoperative hemorrhage or use of pancreatic enzymes post-surgery, especially for the early studies [41]. Risk of bias was assessed at the study, rather than the outcome level due to limited outcome-specific reporting, which may reduce the accuracy of bias judgments for subjective outcomes. First, although the overall rate of POPF was reported in all studies, the lack of stratification of POPF grades across some studies could have led to an underestimate of the occurrence of specific POPF grades. Secondly, pancreatic tumors have a low incidence rate, necessitating a large sample size for clinical trials. The rarity of pancreatic lesions suitable for either EN or resection makes patient recruitment difficult for such trials. Another challenge is the lack of studies reporting long-term morbidity and outcomes like pancreatic insufficiency, recurrence-free survival, and overall survival rates. Heterogeneity among surgeon experience and case volume could have contributed to the rate of morbidity, which could be mitigated by standardized expertise. Furthermore, the heterogeneity in reported outcomes proved to be high in some instances, which can be attributed to differences in patient selection, patient comorbidities, surgeon’s volume/experience, hospital/institution protocols, or available resources across the included studies. Moreover, the lack of reporting the size and location of insulinomas, and the proximity to the MPD in some studies, makes it challenging to determine whether EN was truly indicated. This may have at least partially contributed to an increased incidence of POPF, as evidenced by the high variability in rates, with one study reporting up to 100% POPF rate [36]. Variability between the open approach and minimally invasive techniques, including both laparoscopic and robotic surgery, may have also contributed to the heterogeneity in the rate of POPF [36]. Although the surgical approach was reported in fourteen studies, most did not stratify postoperative outcomes, including POPF, by technique. The few studies that did had limited data, especially in the laparoscopic and robotic groups, making subgroup analysis statistically unreliable. As such, even a limited analysis was not feasible. Future studies should standardize reporting by surgical approach to allow for meaningful comparisons. Additionally, the use of imaging modalities, such as IOUS, which has allowed for better localization and identification of tumors, was not sufficiently reported, which could be a source of bias across studies. Pooling of data regarding the rate and reasoning of lymph node harvesting across the included studies was not feasible. Moreover, the results may not be widely generalizable in populations of patients with a BMI higher than 26.3 which was the mean in this study. Lastly, the evolving guidelines, lack of standardized indications for EN, and heterogeneous reporting of outcomes may have limited the strength of these findings. Therefore, ensuring more homogeneous and robust selection criteria along with standardized reporting of outcomes based on tumor location, distance from the MPD, and surgical approach may enhance and validate the findings from this meta-analysis.

## Conclusion

This review provides precise pooled estimates of key outcomes, including low rates of grade C POPF and recurrence rate, supported by low-to-moderate certainty evidence. Despite these promising results, further studies with standardized reporting, stratification by tumor location and MPD proximity, and longer follow-up are needed to optimize patient selection and confirm long-term safety.

## Supplementary Information

Below is the link to the electronic supplementary material.

Supplementary file1 (DOCX 16 KB)

Supplementary file2 (DOCX 15 KB)

Supplementary file3 (DOCX 223 KB)

Supplementary file4 (DOCX 548 KB)

Supplementary file5 (DOCX 22 KB)

## Data Availability

The dataset used for this meta-analysis will be shared upon request from the study authors.

## Abbreviations

ASA: American society of anesthesiologists
BMI: Body mass index
CT: Computed tomography
DGE: Delayed gastric emptying
DP: Distal pancreatectomy
ERCP: Endoscopic retrograde cholangiopancreatography
EBL: Estimated blood loss
EN: Enucleation
GRADE: Grading of recommendations, assessment, development, and evaluation
IOUS: Intraoperative ultrasound
ISGPS: International study group for pancreatic surgery
MRI: Magnetic resonance imaging
MPD: Main pancreatic duct
MIS: Minimally invasive surgery
NGT: Nasogastric tube
PD: Pancreatoduodenectomy
POAP: Postoperative acute pancreatitis
POD: Postoperative day
POPF: Postoperative pancreatic fistula
PRISMA: Preferred reporting items for systematic reviews and meta-analyses
RFA: Radiofrequency ablation
RCT: Randomized controlled trial
SP: Standardized pancreatectomy
WHO: World Health Organization
