# Supplementary material for: Enucleation for insulinoma: consolidating evidence through systematic review and meta-analysis
Source: Surg Endosc. 2025 Sep 2;39(10):6352–65. doi: 10.1007/s00464-025-12099-0 (PMC12500762; doi:10.1007/s00464-025-12099-0)
Supplement: Supplementary file 1 — Supplementary file1 (DOCX 16 KB) [file 464_2025_12099_MOESM1_ESM.docx]

| **Certainty assessment** | | | | | | **№ of patients** | **Effect** | **Certainty** | **Importance** |
| --- | --- | --- | --- | --- | --- | --- | --- | --- | --- |
| **№ of studies** | **Study design** | **Risk of bias** | **Inconsistency** | **Indirectness** | **Imprecision** | **Enucleation** | **Pooled effect estimate  (95% CI)** |  |  |
| **Overall morbidity** | | | | | | | | | |
| 21 | non-randomized studies | not serious | serious^a^ | not serious | serious^b^ | 277/770 (36.0%) | 37.3% (95%CI: 0.264-0.481) | ⨁⨁◯◯ Low -^a,b^ | IMPORTANT |
| **Recurrence** | | | | | | | | | |
| 17 | non-randomized studies | not serious | not serious | not serious | serious^b^ | 31/646 (4.8%) | 3.1% (95%CI: 0.016-0.045) | ⨁⨁⨁◯  Moderate-^b^ | IMPORTANT |
| **Overall POPF** | | | | | | | | | |
| 17 | non-randomized studies | not serious | serious^a^ | not serious | serious^b^ | 176/652 (27.0%) | 27% (95% CI: 0.179-0.360) | ⨁⨁◯◯ Low -^a,b^ | IMPORTANT |

**CI:** confidence interval

#### Explanations

a. The presence of heterogeneity throughout the main analysis may compromise the consistency of the results.

b. The confidence interval spans priori thresholds for moderate and large effects, limiting certainty in the estimated magnitude.
