# Supplementary material for: Enucleation for insulinoma: consolidating evidence through systematic review and meta-analysis
Source: Surg Endosc. 2025 Sep 2;39(10):6352–65. doi: 10.1007/s00464-025-12099-0 (PMC12500762; doi:10.1007/s00464-025-12099-0)
Supplement: Supplementary file 2 — Supplementary file2 (DOCX 15 KB) [file 464_2025_12099_MOESM2_ESM.docx]

**Supplementary item VI:** Summary of overall postoperative complications after insulinoma enucleation

| ^Post-enucleation complications^ | ^n^  ^(n = 325)^ |
| --- | --- |
| ^Biochemical leak^ | ^84^ |
| ^Grade B pancreatic fistula^ | ^39^ |
| ^Grade C pancreatic fistula^ | ^7^ |
| ^Immediate new-onset diabetes^ | ^4^ |
| ^Pancreatitis^ | ^11^ |
| ^Postoperative bleeding^ | ^10^ |
| ^Abdominal collection^ | ^21^ |
| ^Delayed gastric emptying^ | ^7^ |
| ^Sepsis^ | ^5^ |
| ^Pulmonary complications^ | ^8^ |
| ^Others^ | ^129^ |
