# Supplementary figures and images for: Enucleation for insulinoma: consolidating evidence through systematic review and meta-analysis
Source: Surg Endosc. 2025 Sep 2;39(10):6352–65. doi: 10.1007/s00464-025-12099-0 (PMC12500762; doi:10.1007/s00464-025-12099-0)

**Supplementary item V:** Preferred Reporting Items for Systematic Reviews (PRISMA) Flowchart


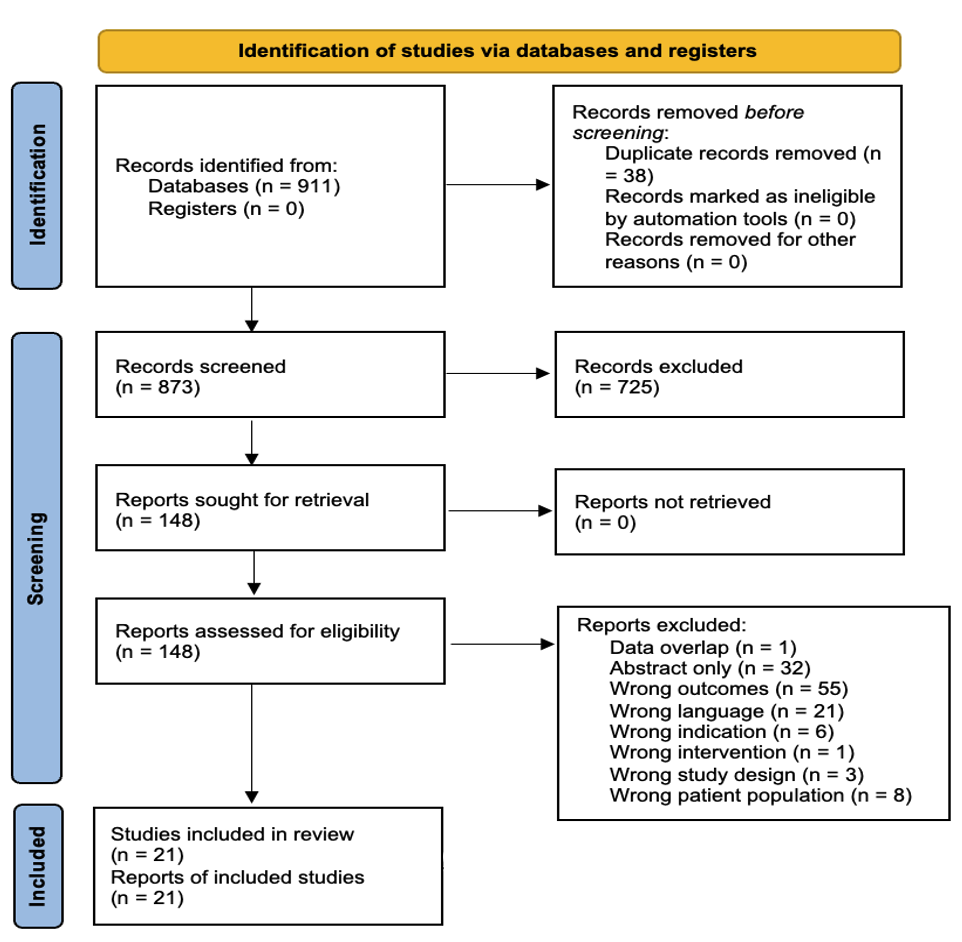

Supplement: Supplementary file 3 — Supplementary file3 (DOCX 223 KB) [file 464_2025_12099_MOESM3_ESM.docx]
