# Supplementary material for: Enucleation for insulinoma: consolidating evidence through systematic review and meta-analysis
Source: Surg Endosc. 2025 Sep 2;39(10):6352–65. doi: 10.1007/s00464-025-12099-0 (PMC12500762; doi:10.1007/s00464-025-12099-0)
Supplement: Supplementary file 4 — Supplementary file4 (DOCX 548 KB) [file 464_2025_12099_MOESM4_ESM.docx]

**Supplementary item IV:** Funnel plots depicting publication bias
