# Supplementary material for: Enucleation for insulinoma: consolidating evidence through systematic review and meta-analysis
Source: Surg Endosc. 2025 Sep 2;39(10):6352–65. doi: 10.1007/s00464-025-12099-0 (PMC12500762; doi:10.1007/s00464-025-12099-0)
Supplement: Supplementary file 5 — Supplementary file5 (DOCX 22 KB) [file 464_2025_12099_MOESM5_ESM.docx]

**Supplementary item II:** Risk of Bias Assessment Using Joanna Briggs Institute Critical Appraisal Checklist for Case Series

| **Study** | Q1 | Q2 | Q3 | Q4 | Q5 | Q6 | Q7 | Q8 | Q9 | Q10 | Total Yes |
| --- | --- | --- | --- | --- | --- | --- | --- | --- | --- | --- | --- |
| **Ayav et al.** | N | N | Y | U | N | N | Y | Y | N | Y | 4 |
| **Belfiori et al.** | Y | Y | Y | Y | Y | N | Y | Y | N | Y | 8 |
| **Chen et al.** | Y | Y | Y | Y | Y | N | Y | Y | N | N | 7 |
| **Chirletti et al.** | Y | Y | Y | Y | Y | N | Y | Y | N | N | 7 |
| **Crippa et al.** | Y | Y | Y | Y | Y | N | Y | Y | N | Y | 8 |
| **Geoghegan et al.** | Y | Y | Y | Y | Y | N | Y | Y | N | N | 7 |
| **Guo et al.** | Y | Y | Y | Y | Y | N | Y | Y | N | Y | 8 |
| **Liu et al.** | Y | Y | Y | U | U | N | Y | Y | N | Y | 6 |
| **Luo et al.** | Y | Y | Y | Y | N | N | Y | Y | N | Y | 7 |
| **Menegaux et al.** | Y | N | Y | Y | Y | N | Y | Y | N | Y | 7 |
| **Naples et al.** | Y | Y | Y | Y | N | Y | Y | Y | Y | Y | 9 |
| **Nikfarjam et al.** | Y | Y | Y | Y | Y | Y | Y | Y | Y | Y | 10 |
| **Peltola et al.** | Y | Y | Y | Y | Y | N | Y | Y | N | Y | 8 |
| **Spelsberg et al.** | N | N | N | Y | Y | N | Y | Y | N | N | 4 |
| **Tsang et al.** | Y | Y | Y | Y | Y | N | Y | Y | N | Y | 8 |
| **vanBeek et al.** | Y | Y | Y | Y | N | N | Y | Y | N | Y | 7 |
| **Vezzosi et al.** | Y | Y | Y | Y | N | N | Y | Y | N | Y | 7 |
| **Wei et al.** | Y | Y | Y | Y | Y | N | Y | Y | N | Y | 8 |
| **Xu et al.** | Y | U | U | Y | Y | N | Y | Y | N | Y | 6 |
| **Yin et al.** | Y | U | U | Y | N | N | Y | Y | N | Y | 5 |
| **Zhang et al.** | U | Y | Y | U | N | N | Y | Y | N | Y | 5 |

Y: Yes, N: No, U: Unclear
